# Adapting an Efficacious Peer-Delivered Physical Activity Program for Survivors of Breast Cancer for Web Platform Delivery: Protocol for a 2-Phase Study

**DOI:** 10.2196/52494

**Published:** 2024-06-19

**Authors:** Bernardine M Pinto, Ashwin Patel, Danielle M Ostendorf, Amy G Huebschmann, Shira I Dunsiger, Madison M Kindred

**Affiliations:** 1 College of Nursing University of South Carolina Columbia, SC United States; 2 Pyx Health Tucson, AZ United States; 3 Department of Medicine University of Colorado Anschutz Medical Campus Aurora, CO United States; 4 Department of Medicine University of Colorado School of Medicine Aurora, CO United States; 5 Brown University School of Public Health Providence, RI United States; 6 University of Augusta Augusta, GA United States

**Keywords:** physical activity adoption, web platform, breast cancer survivors, design, implementation, cost-effectiveness, mobile phone

## Abstract

**Background:**

Interventions promoting physical activity (PA) among survivors of cancer improve their functioning, reduce fatigue, and offer other benefits in cancer recovery and risk reduction for future cancer. There is a need for interventions that can be implemented on a wider scale than that is possible in research settings. We have previously demonstrated that a 3-month peer-delivered PA program (Moving Forward Together [MFT]) significantly increased the moderate to vigorous PA (MVPA) of survivors of breast cancer.

**Objective:**

Our goal is to scale up the MFT program by adapting an existing peer mentoring web platform, Mentor1to1. InquistHealth’s web platform (Mentor1to1) has demonstrated efficacy in peer mentoring for chronic disease management. We will partner with InquisitHealth to adapt their web platform for MFT. The adaptation will allow for automating key resource-intensive components such as matching survivors with a coach via the web-based peer mentoring platform and collecting key indexes to prepare for large-scale implementation. The aim is to streamline intervention delivery, assure fidelity, and improve survivor outcomes.

**Methods:**

In phase 1 of this 2-phase study, we will interview 4 peer mentors or coaches with experience in delivering MFT and use their feedback to create Mentor1to1 web platform adapted for MFT (webMFT). Next, another 4 coaches will participate in rapid, iterative user-centered testing of webMFT. In phase 2, we will conduct a randomized controlled trial by recruiting and training 10 to 12 coaches from cancer organizations to deliver webMFT to 56 survivors of breast cancer, who will be assigned to receive either webMFT or MVPA tracking (control) for 3 months. We will assess effectiveness with survivors’ accelerometer-measured MVPA and self-reported psychosocial well-being at baseline and 3 months. We will assess implementation outcomes, including acceptability, feasibility, and program costs from the perspective of survivors, coaches, and collaborating organizations, as guided by the expanded Reach, Effectiveness, Adoption, Implementation, Maintenance (RE-AIM) framework.

**Results:**

As of September 2023, phase 1 of the study was completed, and 61 survivors were enrolled in phase 2. Using newer technologies for enhanced intervention delivery, program management, and automated data collection has the exciting promise of facilitating effective implementation by organizations with limited resources. Adapting evidence-based MFT to a customized web platform and collecting data at multiple levels (coaches, survivors, and organizations) along with costs will provide a strong foundation for a robust multisite implementation trial to increase MVPA and its benefits among many more survivors of breast cancer.

**Conclusions:**

The quantitative and qualitative data collected from survivors of cancer, coaches, and organizations will be analyzed to inform a future larger-scale trial of peer mentoring for PA delivered by cancer care organizations to survivors.

**Trial Registration:**

ClinicalTrials.gov NCT05409664; https://clinicaltrials.gov/study/NCT05409664

**International Registered Report Identifier (IRRID):**

DERR1-10.2196/52494

## Introduction

### Background

The 5-year survival rates for female survivors of breast cancer have increased from 63% in the 1960s to 90% currently, with >3.6 million survivors of breast cancer in the United States today [1]. There is a large body of evidence on the benefits of aerobic exercise to alleviate sequelae of cancer treatments when conducted with onsite supervision, as well as home-based programs [2-6]. Hence, the American College of Sports Medicine [7] and the American Cancer Society (ACS) [8] recommend that survivors participate in aerobic exercise at moderate to vigorous intensity for at least 150 minutes per week. However, only 24% to 44% of survivors meet these guidelines [8].

The literature suggests that leveraging peer support may help improve moderate to vigorous physical activity (MVPA) adoption among survivors of breast cancer [9]. Sociocultural and communication theories suggest that people are more receptive to assistance when it is delivered by someone perceived as similar to oneself (eg, of comparable age and life experiences) [10]. Peer support for health is used to maximize impact, sustainability, and scale-up of successful interventions while facing limited resources and contextual constraints. With the growing evidence of the key role that physical activity (PA) can play in cancer recovery, we are one of the first teams to extend these research findings into the “real world” by training community volunteers to encourage survivors to adopt MVPA [11-13].

We chose to test the dissemination potential at the ACS, a not-for-profit community-based organization. Specifically, ACS’ Reach to Recovery peer mentors provide emotional support and information to patients with cancer. Hence, it was a “natural fit” to test the effects of these peer coaches encouraging sedentary survivors to become more physically active to enhance their recovery from cancer treatment. Our prior randomized controlled trial (RCT) showed that this peer-delivered intervention (Moving Forward Together [MFT]) was effective in increasing survivors’ MVPA [12]. Coaches who were trained and supervised to provide MVPA counseling via telephone calls to other survivors of breast cancer effectively helped them significantly increase their MVPA to a mean of 129 minutes per week at 12 weeks (*P*<.05).

The telephone counseling component of our evidence-based MVPA intervention is based on the Transtheoretical Model (TTM) [14,15] and Social Cognitive Theory (SCT) [16] of behavior change that have been applied to exercise behavior. Key constructs from the TTM (motivational readiness) and SCT (self-efficacy and beliefs about the importance of MVPA in reducing fatigue etc) underlie the telephone counseling that coaches delivered to survivors in our prior work [12]. We plan to extend these individual-level theories with frameworks from the field of dissemination and implementation science to assess contextual and organizational-level factors that are critical to planning for large-scale implementation of our evidence-based intervention. We will use the expanded Reach, Effectiveness, Adoption, Implementation, Maintenance (RE-AIM) framework of implementation outcome dimensions that includes a model of key contextual determinants of successful implementation, that is, the Practical, Robust Implementation and Sustainability Model (PRISM) [17,18]. RE-AIM has been in use since 1999 and has been applied widely to plan and evaluate the translation of many interventions in primary care and community settings, including smoking cessation interventions, diabetes management, and MVPA promotion [19,20]). As PRISM operationalizes the organizational staff and survivor domains that influence implementation success, we will use PRISM to identify contextual and organizational factors that influence RE-AIM outcomes [18].

### Objective

To address the gap in the literature of limited real-world translation of MVPA promotion among survivors of cancer, our study objective is to develop and test the implementation of Mentor1to1 web platform adapted for MFT (webMFT) as a scalable intervention to effectively increase MVPA among survivors of breast cancer. The study has 2 phases: phase 1, which includes aims 1 and 2, and phase 2 which includes aim 3.

#### Phase 1

In phase 1, we will work with peer coaches who have prior experience in delivering MFT to adapt an existing web platform, Mentor1to1 developed by InquisitHealth Inc (a technology company that offers peer mentoring for chronic disease management via a web platform). In aim 1, we will conduct semistructured interviews with 3 to 4 peer coaches who have already delivered MFT in our prior work to obtain their feedback on how to adapt Mentor1to1 for MFT. In aim 2, we will use the interview data and use rapid, iterative user-centered design principles with another 3 to 4 peer coaches (with prior experience with MFT) to create webMFT.

#### Phase 2

For phase 2, the study aims are as follows:

- Aim 3a: conduct an RCT wherein 10 to 12 peer coaches will be trained to deliver webMFT to 56 survivors of breast cancer, who will be randomly assigned to receive either webMFT or MVPA tracking. We will collect quantitative and qualitative data on the acceptability and feasibility of webMFT (eg, number and duration of calls delivered and barriers experienced) and suggestions for improvement from the coaches.
- Aim 3b: obtain data on survivor outcomes at 12 weeks from accelerometer-measured MVPA (primary outcome) and self-reported quality of life (QOL), physical functioning, mood, and fatigue to help estimate effect sizes for a larger trial to test the impact of webMFT versus MVPA tracking on survivor effectiveness outcomes. We expect that at the postintervention time point, survivors randomized to webMFT will increase MVPA; improve QOL, physical functioning and mood; and decrease fatigue significantly more than survivors randomized to MVPA tracking.
- Aim 3c: assess organizational-level factors (eg, the barriers and facilitators for implementation, setting and contextual factors, resources required, and alignment of webMFT with workflow and mission) using the RE-AIM and PRISM models [18] by interviewing key multilevel stakeholders at the collaborating organizations.
- Aim 3d: collect detailed cost, time, and resource information to conduct cost-effectiveness analyses from the perspectives of the survivors, coaches, and cancer care organizations.
- Aim 3e: develop a guide on how to recruit and build collaborations with other organizations that offer peer mentor programs with which webMFT can be integrated. The long-term goal of this work is to use these data to inform a robust, large-scale pragmatic trial with several cancer care organizations to deliver webMFT to survivors of breast cancer across the country.

## Methods

### Study Design

The study will consist of 2 phases: phase 1 will involve the adaptation and user-centered testing of Mentor1to1 for MFT. We will recruit coaches from past studies who have previously delivered the MFT program. These coaches have experience with intervention content and delivery and thus will be able to provide informed feedback. In phase 2, we will test webMFT using a randomized controlled design.

### Phase 1

#### Recruitment of Coaches

Peer mentors who participated as coaches for the MFT program in a prior RCT [13] will be invited to participate. The eligibility criteria are that the participants must: (1) be aged ≥21 years, (2) currently have an email address and telephone access, and (c) have access to a tablet or computer with internet. We anticipate recruiting 6 to 8 coaches.

#### Adaptation of the Web Platform (Mentor1to1) for MFT

This includes (1) customizing coach-survivor matching criteria; (2) defining the MFT program path in terms of call frequency and counseling content; (3) counseling questions that coaches will ask survivors during each call; (4) counseling questions to structure data entry for coaches via the web platform, and (5) integration of a Fitbit tracker (Fitbit Inc) to autocapture MVPA, heart rate, and step data. The coach-survivor’s matching criteria, progression in the 12-week program, counseling content, and review of the survivor’s weekly MVPA data are based on our prior studies [12,13] and interview data collected during the principal investigator’s participation in the National Cancer Institute’s Speeding Research-Tested Interventions program.

The steps involved in the development process is as follows: the web platform will be rapidly and iteratively tested by (1) InquisitHealth’s team to ensure navigational ease and functionality robustness and (2) our research team to ensure accuracy regarding our design intentions and completeness and fidelity to the content and process of MVPA counseling within MFT. (3) InquisitHealth will conduct interviews via Zoom with 3 to 4 coaches who will complete usability tasks (eg, creating an account in webMFT). (4) After incorporating all available feedback, an additional 3 to 4 coaches (with prior MFT experience) will review the entire system and provide qualitative and quantitative feedback. Quantitative feedback will include the System Usability Scale, which consists of 10 items where coaches will rate the usability of webMFT using a Likert scale (1=strongly disagree; 5=strongly agree) [21]. System Usability Scale has been shown to be reliable and valid. On the basis of recommendations from the coaches, the InquisitHealth team will refine webMFT. Hence, we will adhere to well-established iterative user-centered design principles in adapting Mentor1to1 for evidence-based MFT [22-24]. Coaches will receive a US $100 gift card for their participation.

### Phase 2

**Figure 1:**
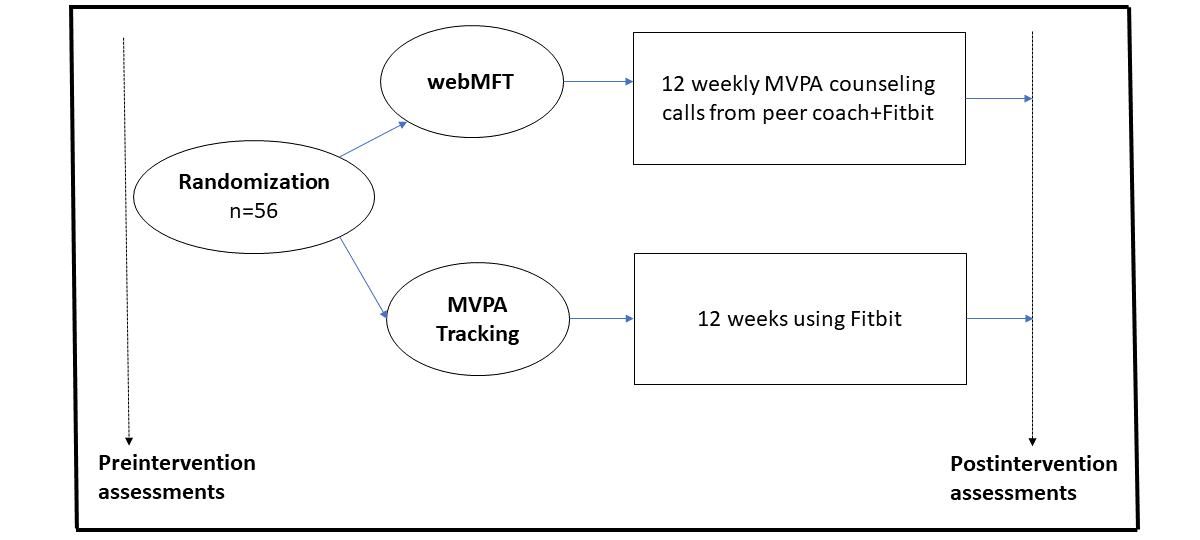
Study design (phase 2). MVPA: moderate to vigorous physical activity; webMFT: Mentor1to1 web platform adapted for Moving Forward Together.

Phase 2 will be an RCT of webMFT wherein 10 to 12 coaches (naive to the MFT program, across the collaborating cancer care organizations that have peer mentoring programs) will be trained to deliver webMFT to survivors of breast cancer. We will recruit 56 survivors of breast cancer and randomize them to webMFT (assigned a coach to deliver intervention via the web platform) or an MVPA tracking group (independent MVPA tracking; refer to Figure 1). We will use multiple methods to examine the acceptability and feasibility of the coaches using webMFT and to gather data on the implementation and effects of webMFT on survivors’ MVPA (primary outcome), mood, QOL, physical functioning, and fatigue at baseline and 12 weeks. Finally, we will conduct individual interviews with multilevel stakeholders at the collaborating organizations to collect data, based on RE-AIM outcome dimensions and PRISM domains, to guide the implementation of webMFT on a larger scale.

### Description of webMFT

Mentor1to1 is a web-based platform for coach and survivor enrollment; survivor-to-coach matching; one-on-one remote coach mentoring via phone, text, and smartphone messaging; automated MVPA tracking via Fitbit; and administrator oversight and support. Access to the system by both the coach and staff is password protected. Within the webMFT, the coach will be able to review information from prior calls (eg, a survivor’s MVPA recorded by a Fitbit activity tracker) and review information for the upcoming call (eg, counseling questions). When ready for a call to a webMFT participant, the coach will click a button on the computer to initiate the call. The system calls the survivor and connects the coach and survivor for a one-on-one telephone conversation. This avoids the need to exchange contact information. All calls are generated from a secure server, allowing the tracking of call frequency, length, and recording for quality control, safety, and training purposes, while respecting Health Insurance Portability and Accountability Act (HIPAA)–mandated security and privacy requirements. During the call, coaches will be expected to document their discussion for each specified counseling question; additional notes can be taken throughout the call within the platform. At the end of each call, coaches will collaboratively set MVPA goals with the participants, discuss strategies to achieve those goals in the upcoming week, and schedule the next call. As needed, the platform will assist with scheduling time zone differences between the coach and the participant. The system will send email or text reminders to the coach and the participant about their upcoming call.

### Recruitment of Coaches

We will work with cancer care organizations (eg, Cancer Hope Network and Pink Lemonade Project) to recruit coaches and survivors. In total, we will recruit 10 to 12 coaches across the organizations to be trained to deliver webMFT. Each coach will be asked to work with 4 to 5 survivors over the study duration. As in our prior work [12,13], the organizations will mail or email informational letters and brochures to peer mentors, offering them the opportunity to become a coach and receive training in the delivery of webMFT.

Eligibility criteria for coaches include that they must (1) be a current peer mentor (at least 1 year) at the organization; (2) have an email address, telephone access, and access to a tablet or computer with internet to use the web platform; (3) be willing to participate in group training via videoconference; (4) be willing to have calls recorded and reviewed; (5) be willing to be supervised; (6) have a baseline level of digital literacy for this health technology (ie, sufficient internet speed and proficient in typing); and (7) currently exercise for at least 60 minutes per week.

### Survivor Recruitment

The collaborating cancer care organizations will be asked to send informational mailings or emails about the study to their lists consisting of survivors of breast cancer. Other recruitment avenues will include informational mailings from oncology practices, tumor registries, and community events. Interested survivors will be asked to contact the study’s research assistant who will explain the study and obtain verbal informed consent to conduct a telephone screen for eligibility. If eligible, the staff will send the survivor an informed consent document through REDCap (Research Electronic Data Capture; Vanderbilt University) [25,26]; once signed, participants will be sent a copy for their records. Participants will be mailed a physician consent form for their participation in the study. After the consent forms are obtained, all survivors will be mailed an Actigraph (GT3X; Actigraph LLC) accelerometer to monitor MVPA with instructions and a stamped envelope to return the Actigraph. In addition, they will receive instructions on completing baseline questionnaires within REDCap. After the baseline assessments are completed and the Actigraph has been reviewed for wear time and confirmation of a sedentary lifestyle, participants will be mailed a Fitbit Inspire 2 tracker. Participants will also receive a Fitbit manual and a study username and password to log in to the Fitbit app on their phone. Survivors randomized to the MVPA tracking arm will receive an identical Fitbit in the mail; their data will be tracked directly via the Fitbit Application Programming Interface.

Eligibility criteria for survivors include the following: (1) women aged >21 years; (2) diagnosis in the past 5 years with stage 0 to stage 3 breast cancer (completed surgery, radiation, or chemotherapy, and those currently receiving hormone therapy; physician consent for all study survivors will be obtained); (3) ability to read and speak English; (4) those with sedentary lifestyle (ie, <30 minutes per week of vigorous exercise or <90 minutes per week of moderate intensity exercise for the past 6 months); (5) ability to walk unassisted; and (6) access to a smartphone with Bluetooth and internet access. Women with more advanced disease (stage 4) and medical or psychiatric problems (eg, coronary artery disease, peripheral vascular disease, diabetes, and orthopedic problems) that may interfere with protocol adherence will not be included. These inclusion and exclusion criteria are similar to those used in our prior work [12,13]. Survivors will be asked to provide consent for medical chart review to extract disease and treatment history.

### Ethical Considerations

Phase 1 of the study was deemed exempt by the institutional review board of the University of South Carolina (Pro00117242), and phase 2 of the study was approved by the same institutional review board. All study participants and coaches will provide written informed consent to participate in the study.

Study data will be deidentified before analyses and dissemination.

Coaches taking part in phase 1 of the study will be provided with US $100 as an incentive. Coaches taking part in phase 2 will be paid US $30 upon completion of training. In addition, coaches will be provided US $25 on delivering the intervention to their first participant and US $25 during the holiday season. Study participants (survivors) in phase 2 will be paid US $25 for completing baseline assessments and US $25 for assessments at the postintervention time point.

### Sample Size

Power calculations were based on data from our prior work with MFT [27], and data from the study by Cadmus-Bertram et al [28], who compared web-based tracking+Fitbit to pedometer alone among postmenopausal women. Comparing the effects of coach counseling [27] to web-based tracking+Fitbit [28] on changes from baseline in MVPA would yield a medium effect size, *d*=0.60. Assuming similar effect sizes and a 2-sided α=.05, we would have more than sufficient power to test intervention effects on changes in MVPA (minutes per week) at the end of intervention with 20 survivors per arm. However, given the risk of powering on pilot studies, we have conservatively inflated the sample size to 25 survivors per arm. On the basis of our prior work, we expect attrition to be ≤10% at 12 weeks, so we will recruit a total of 56 women (n=28 survivors per arm) to obtain complete data from 50 survivors.

### Study Implementation

#### Survivor Enrollment

Each survivor will be asked to obtain medical consent from her oncologist to enroll. Providers will be allowed to exclude patients if the MVPA goal would be unsafe for the patient. Intervention delivery will begin after informed consent, medical consent, and baseline data are obtained.

#### Random Assignment

Survivors will be randomly assigned to a group after informed consent and baseline data are obtained. Consistent with prior work, the sample will be stratified for 2 variables that may affect MVPA outcomes (age: <60 years or >60 years; and treatment: received or not received chemotherapy). The randomization scheme will be generated using the R (the R Foundation for Statistical Computing), based on a permuted block randomized procedure with small, random-size blocks so that equal numbers of survivors are randomized to webMFT or MVPA tracking by age and treatment. The project director will contact the survivor and disclose the group assignment from a sealed envelope.

#### Matching

The coach-survivor matching criteria for survivors randomized to webMFT will include mandatory and preference-driven criteria. The mandatory criterion is overlapping times on their respective schedules. The preference-driven criteria include age (+10 years or −10 years), type of cancer treatment (eg, radiation), race, and ethnicity. If >1 matching coach exists, preference-driven criteria will be used to identify an appropriate coach. Following matching, the first call between a coach and a survivor will be scheduled. The coach will receive an alert (email and text, depending on preference) that a new survivor has been added to the queue.

#### The webMFT Group

Survivors randomized to webMFT will receive the evidence-based MFT program for MVPA promotion [29] that consists of MVPA counseling matched to patients’ motivational readiness and self-monitoring of MVPA (Fitbit activity tracker).

#### Exercise Counseling

Coaches will be asked to contact survivors using the web platform and make 12 calls (1 call per week over 12 weeks). Each call will take approximately 15 to 20 minutes. The purpose of the calls is to build a supportive relationship with the survivor, assess motivational readiness, review MVPA participation (participant Fitbit data viewed on the web platform), identify any health concerns, assist the survivor to identify relevant barriers to MVPA, and help her to problem solve such barriers and set MVPA goals. In addition, the coach will provide feedback and reinforce and encourage efforts to start being active and stay active. The counseling will be matched to the survivor’s motivational readiness assessed at the start of each call. For example, women in the precontemplation stage will be given information to increase their awareness of the benefits of MVPA after cancer treatments (eg, improved physical functioning). The goal, as in prior work, will be to gradually increase the amount of moderate intensity aerobic exercise that is performed to the current national recommendations of at least 150 minutes of MVPA per week [7]. Coaches will be trained not to provide medical advice but to encourage patients to contact their oncologist for medical care and health issues. If patients report symptoms such as chest pain or difficulty breathing, participation will be temporarily halted, and they will be referred to their oncologist.

#### Self-Monitoring of MVPA

Survivors will be asked to self-monitor MVPA participation (frequency, duration, steps, and heart rate during MVPA) by wearing the Fitbit activity tracker each day. These data will be automatically uploaded to the webMFT platform via the Fitbit Application Programming Interface integrated into the Mentor1to1 platform. Participants will be encouraged to sync their Fitbit daily via Bluetooth. Automated reminders to sync the tracker will be sent from the web platform. Coaches will be able to email or text the participant their Fitbit data (chiefly, active minutes) as shown on the web platform. Coaches will review their survivor’s weekly MVPA during calls and enter these data into the web platform.

#### MVPA Tracking Group

These survivors will be asked to self-monitor MVPA participation (frequency, duration, and heart rate during MVPA) by wearing the Fitbit device each day over 12 weeks (3 months). They will be able to see the feedback that the tracker provides on the app dashboard (eg, daily MVPA minutes) and can access other Fitbit features. They will receive weekly text reminders from the research staff members to sync the tracker. The research staff members will review Fitbit data through the Fitbit dashboard to ensure that the device is worn and routinely synced. This group will be provided with the same 12 tip sheets as those provided to webMFT group and the procedures to follow in the event of any injury. This comparison arm thus constitutes a real-world, low-cost intervention.

#### Training Coaches

Before beginning training, coaches will receive a training manual. On the basis of prior work, we will develop recorded training modules that provide information on the benefits of exercise for survivors of cancer; TTM and SCT; training on counseling techniques (eg, empathy and reflective listening); working with human participants and HIPAA regulations; and emergency protocols. In addition, trainers will offer 1 live internet-based group training session during which coaches will practice role plays and receive feedback through videoconferencing. Coaches will complete a second live internet-based individual training session to ensure that they can deliver the intervention while using the platform, using screen sharing capabilities between the coach and research staff. Coaches will be trained to query patients at each call about health symptoms and notify the research staff immediately if survivors endorse any symptoms that are potentially indicative of a serious problem. In these cases, study participation will be temporarily halted until the medical issue has been resolved. Coaches will receive a supervision call every other week from research staff members through the web platform.

### Measures

#### Overview

In phase 2, we will collect RE-AIM outcome measures from survivors, coaches, and organizational staff members as well as key contextual and organizational factors that influence RE-AIM outcomes using PRISM (Tables 1-3). Quantitative and qualitative data will be collected. Perspectives from the survivors, coaches, and organizational staff members will be obtained through semistructured interviews. Interview guides will be developed based on the RE-AIM outcome dimensions and PRISM domains of contextual influence. The RE-AIM outcome dimensions assessed include Effectiveness, as well as the implementation outcomes of Reach, Adoption, Implementation, and Maintenance. The PRISM domains include: (1) perspective of the intervention at the level of the survivor, coach and organization; (2) organizational characteristics; (3) external environment; and (4) implementation and sustainability infrastructure [17,18].

**Table 1:** Quantitative data collection for effectiveness outcomes.

Effectiveness outcome	Measure	Participant level	Time point	
		Survivors	Baseline	12 weeks
MVPA^a^	Accelerometer (Actigraph GT3X) worn over 7-d period	✓	✓	✓
QOL^b^	36-item Short Form Health Survey	✓	✓	✓
Physical functioning	FACT-B^c^	✓	✓	✓
Fatigue	FACT-F^d^	✓	✓	✓
Mood	POMS^e^	✓	✓	✓
Stages of change	Stage of Readiness for Exercise	✓	✓	✓
Self-efficacy for exercise	Exercise Self-Efficacy Scale	✓	✓	✓

^a^MVPA: moderate to vigorous physical activity.

^b^QOL: quality of life.

^c^FACT-B: Functional Assessment of Cancer Therapy Scale-Breast.

^d^FACT-F: Functional Assessment of Cancer Therapy Scale-Fatigue.

^e^POMS: Profile of Mood States.

**Table 2:** Quantitative data collection for reach, adoption, and implementation outcomes.

RE-AIM^a^ dimension and PRISM^b^ domain outcome			Measure	Participant level			Time point	
				Survivor	Coach	Organization	Baseline	12 weeks
**Reach**								
	Total potential survivors in participating organizations		Count	✓			✓	
	Number of survivors eligible to participate (meet study eligibility criteria)		Count, % of total	✓			✓	
	Number of survivors excluded by the investigator due to not meeting eligibility criteria for study		Count, % of total	✓			✓	
	Reasons survivors excluded by the investigator		Descriptive data tracked by the study team	✓			✓	
	Number of eligible survivors who agree to enroll		Count, % of eligible	✓			✓	
	Number of eligible survivors who decline		Count, % of eligible	✓			✓	
	Reasons eligible survivors decline		Tracked by study team	✓			✓	
**Adoption**								
	Total number of potential settings		Count			✓	✓	
	Number of settings eligible (based on organizational readiness criteria)		Count, % of total			✓	✓	
	Number of settings excluded by investigator (based on organizational readiness criteria)		Count, % of total			✓	✓	
	Reasons settings excluded by the investigator		Settings score <4 on ORIC^c^ questions or <4 on single-item organizational readiness survey or informed study team not ready or PI^d^ discretion			✓	✓	
	Number of eligible settings that agree to participate in MFT^e^		Count, % of eligible			✓	✓	
	Number of eligible settings that decline to participate in MFT		Count, % of eligible			✓	✓	
	Reasons eligible settings choose to agree to participate or decline to participate in MFT		Open-ended item at the end of ORIC survey			✓	✓	
	Number of eligible settings not contacted or other		Count, % of eligible			✓	✓	
	Setting: characteristics of adopters vs nonadopters		Number of employees, location, etc			✓	✓	
	Within each site, number of coaches who agree to participate in MFT		Count, % within a participating setting		✓		✓	
	Within each site, number of coaches who decline to participate in MFT		Count, % within a participating setting		✓		✓	
	Reasons coaches choose to decline to participate in MFT		Tracked by the study team		✓		✓	
	Coaches: characteristics of adopters vs nonadopters		Age, sex, race, ethnicity, and baseline activity levels		✓		✓	
**Implementation**								
	**Perspective of webMFT^f^ intervention^g^**							
		Acceptability of webMFT	Study-specific questionnaire IAM^h^ (4-items) NPS^i^	✓	✓			✓
		Feasibility of webMFT	FIM^j^; 2-items: is webMFT easy to use? Doable?	✓	✓			✓
	Adaptations^k^		FRAME^l^		✓			✓
	Costs^k^		Cost tracking form	✓	✓			✓
	Fidelity^k^		Auditing of coach-delivered calls		✓			✓
	**Patient characteristics**							
		Patient demographics	Age, race, ethnicity, baseline level of MVPA^m^, cancer treatment status, and home address	✓			✓	
	**Organizational characteristics**							
		Organizational readiness	ORIC			✓	✓	

^a^RE-AIM: Reach, Effectiveness, Adoption, Implementation, Maintenance

^b^PRISM: Practical, Robust Implementation and Sustainability Model

^c^ORIC: Organizational Readiness for Implementing Change.

^d^ PI: principal investigator.

^e^MFT: Moving Forward Together.

^f^webMFT: Mentor1to1 web platform adapted for Moving Forward Together.

^g^Practical, Robust Implementation and Sustainability Model (PRISM) domains.

^h^IAM: Intervention Appropriateness Measure.

^i^NPS: Net Promoter Score.

^j^FIM: Feasibility of Intervention Measure.

^k^These measures will be tracked continuously throughout the study.

^l^FRAME: Framework for Reporting Adaptations and Modifications-Enhanced.

^m^MVPA: moderate to vigorous physical activity.

**Table 3:** Qualitative data collection for Reach, Effectiveness, Adoption, Implementation, Maintenance (RE-AIM) and Practical, Robust Implementation and Sustainability Model (PRISM).

RE-AIM dimension, PRISM domain, and outcome				Description (interview^a^ questions)		Participant type				
						Survivors		Coaches		Organizational staff
**Reach**										
	**Perspective of webMFT^b^ intervention, survivor characteristics**									
		Motivation	What factors did you consider when you agreed to participate in webMFT? What motivated you, if anything, to stay in the intervention for the full 12 weeks? If you withdrew, why?		✓					
**Effectiveness**										
	**Perspective of webMFT intervention**									
		Program value	How did webMFT impact health and well-being of the survivors? What have you learned from participating in webMFT? How might webMFT benefit you?		✓		✓			
**Adoption**										
	**Perspective of webMFT intervention, Organizational Characteristics**									
		Motivation	What factors did you consider when you agreed to participate in webMFT? What motivated you to continue volunteering with webMFT?				✓		✓	
**Implementation**										
	**Perspective of webMFT intervention**									
		Acceptability of webMFT	Is webMFT acceptable? Did you like the intervention? What went well? What did not go well?		✓		✓		✓	
		Feasibility of implementing webMFT	Was webMFT feasible? What went well? What didn’t go well? Suggestions for improvements		✓		✓		✓	
		Patient centeredness	How did the intervention align (or not align) with the needs?		✓		✓		✓	
		Adaptations	What changes were made to webMFT during implementation?				✓		✓	
		Organizational readiness	How ready were you to implement webMFT?				✓		✓	
		Burden	Time and costs of implementation, unintended effects from webMFT		✓		✓		✓	
		Program fit	How does webMFT fit with your values and mission?						✓	
	**Organizational characteristics**									
		Climate and culture	Shared goals with webMFT; alignment with mission				✓		✓	
		—^c^	Data and decision support; workflow				✓		✓	
		Organizational readiness	Capacity to partner; What factored into your decision to partner to implement webMFT? What would have made it easier or harder to partner?						✓	
	**External environment**									
		—	Community resources, apps, local policies, access to safe places to recreate, urban or rural, access to Wi-Fi, insurance reimbursement, etc		✓		✓		✓	
	**Implementation infrastructure**									
		Implementation strategies	Coach training and support, resources, tools or skills Inclusion of webMFT roles into job descriptions and performance evals				✓		✓	
**Maintenance**										
	**Sustainability infrastructure**									
		Potential for sustainability	Intention to maintain webMFT; factors that would make it more sustainable (cost of delivery)				✓		✓	
		Motivation to sustain webMFT	Intention to continue, discontinue, or adapt webMFT delivery (coaches and staff); motivation to continue exercise behavior (survivors)		✓		✓		✓	

^a^Interviews will last approximately 45 to 60 minutes.

^b^webMFT: Mentor1to1 web platform adapted for Moving Forward Together.

^c^Not applicable.

#### Effectiveness

Intervention effectiveness, operationalized as changes in survivors’ MVPA (primary outcome), will be assessed at the postintervention time point (3 months; Table 1).

Postintervention assessments will follow the same procedures as at baseline: the questionnaires will be completed via REDCap and staff members conducting assessments will be blinded to the survivors’ group assignments. Staff members will mail the accelerometer to the patients. When the units are returned by mail and web-based questionnaires completed, survivors will receive a US $25 gift card (US $25×2 assessments=US $50). The assessments are described in Tables 1 and 3 and include the following:

1. Accelerometer: we will use the Actigraph accelerometer (GT3X) as a device-based and gold standard measure of MVPA. Survivors will be asked to wear the unit around their waist during all waking hours over a 7-day period. The Actigraph will be worn at baseline and the postintervention time point. The Freedson cut points [30] will be used to score the data; data will be collected in 10-second epochs. A valid accelerometer day is defined as >10 hours per day; participants are required to have 4 valid days of wear.
2. QOL, physical functioning, fatigue and mood will be assessed using validated and standardized questionnaires that have been used by patients with breast cancer. Survivors will be asked to complete the following standardized questionnaires via REDCap: Functional Assessment of Cancer Therapy Scale-Breast [31], a 36-item Short Form Health Survey that includes a Physical Functioning subscale [32], Functional Assessment of Cancer Therapy Scale-Fatigue [33], and the Profile of Mood States [34]. In addition, the following questionnaires will be used to assess the constructs relevant to the TTM and SCT: Stage of Readiness for Exercise [35] and Exercise Self-Efficacy [36].
3. Program value will be assessed during semistructured interviews at the study end with survivors to understand how webMFT impacted the health and well-being of the survivors (Table 3).

#### RE-AIM Implementation Outcomes

##### Reach

Reach will be assessed at the level of the survivor and will include the number of survivors eligible to participate out of the total potential survivors at participating organizations as well as the number of survivors excluded [18,37]. Furthermore, survivors will be asked about factors that influenced their motivation to join the program during semistructured interviews conducted at the postintervention time point (refer to Tables 2 and 3 for additional details).

##### Adoption

Adoption will be assessed at the level of the organization and the level of the coach and will include the total number of eligible organizations out of the total number of potential organizations, the percentage of eligible organizations that agree to participate in the study, as well as the percentage of eligible coaches who agree to participate to deliver webMFT [18,37]. In addition, coaches and organizational staff will be asked about factors that influenced their decision to participate in the study during semistructured interviews (refer to Tables 2 and 3 for additional details).

##### Implementation

On the basis of RE-AIM, the implementation dimension includes measures of the costs of delivering webMFT, as well as the fidelity of delivery and adaptations needed to feasibly deliver the program in an acceptable way to end users [18]. Accordingly, we have also grouped our assessment of feasibility and acceptability outcomes in this RE-AIM dimension, as well as the assessment of organizational readiness that relates to the feasibility of program delivery.

Implementation outcome measures of acceptability and feasibility of webMFT will be assessed at the postintervention time point (3 months). In addition, we will track adaptations to webMFT, costs of delivering webMFT, and fidelity to the program throughout the implementation of webMFT. Organizational readiness will be assessed at the level of the organization. Finally, we will collect several qualitative assessments of additional implementation outcomes based on the PRISM domains [18], through semistructured interviews with survivors, coaches and organizational staff members (Table 3). These assessments are described in Tables 2 and 3 and include the following:

1. Acceptability and feasibility: at 12 weeks, the survivors randomized to webMFT group and the coaches will evaluate the intervention components (eg, usefulness of calls and satisfaction) via a REDCap questionnaire [29,38] and during semistructured interviews. At the end of their study participation, all coaches will complete an evaluation (using REDCap) of the components of webMFT using a feedback questionnaire similar to the one used in prior trials, with additional items that focus on the web platform. Coaches will complete evaluations (on a 1-4 rating scale, 1=not at all satisfied; 4=very satisfied) of satisfaction and acceptability of each webMFT component (eg, viewing patient’s MVPA) in REDCap. We will also evaluate the number and duration of the calls delivered (tracked by the web platform) and barriers identified by the coaches on the feedback questionnaire. Furthermore, both survivors and coaches will complete additional quantitative measures including the 4 items from the Intervention Appropriateness Measure [39], 2 items from the Feasibility of Intervention Measure—is the intervention (1) easy to use and (2) doable? [39]—and the Net Promoter Score [40,41]. During intervention delivery, coaches will be interviewed to explore their experiences with webMFT and to elicit suggestions for improvements. These data will be used to modify webMFT at the end of the study.
2. Adaptations: adaptations to webMFT will be assessed at the level of the coach and the level of the organizational staff. We will track adaptations throughout the duration of the implementation of webMFT based on a survey adapted from the Framework for Reporting Adaptations and Modifications-Enhanced (FRAME; Table 2) [42] and using qualitative methods (Table 3).
3. Costs: we will assess patient costs as in prior studies [27] to capture out-of-pocket expenses and any health care costs related to MVPA. We will also assess the costs for coach training or intervention delivery, using established time-based activity costing techniques [43]. We will use the quantitative data to inform the development of debrief questions and recontact a subgroup of webMFT survivors (n=20, randomly selected) and conduct interviews to understand the patient perspective of webMFT and elicit suggestions for improvements, based on RE-AIM dimensions and PRISM domains. The time taken by the coaches to deliver webMFT (ie, for preparation, delivery, and follow-up of calls) will be tracked on the web platform and during biweekly calls with the project director. These data along with the time spent in training and supervision will allow us to estimate costs to the coaches, a key issue in designing for dissemination [44].
4. Fidelity: intervention fidelity will be assessed using auditing of the coach-delivered calls as has been done in prior work [11-13]. The principal investigator will audit 10% of completed coach calls throughout the duration of implementation of webMFT.
5. Organizational readiness: we will conduct brief surveys (assessing demographics and prior experience with research collaborations) and telephone-based interviews with key individuals in each partner organization. Each potential organization will also be asked to complete the Organizational Readiness for Implementing Change questionnaire [45]. We anticipate interviewing 4 to 6 individuals (1-2 per organization) such as the peer supervisor. Semistructured interviews will be scheduled at a time that is convenient for the interviewee, will last approximately 45 to 60 minutes, and will be audio recorded. A research team member will conduct the interviews and use a semistructured interview guide to ensure that each interviewee is asked the same core questions, based on PRISM domains.

##### Maintenance

Potential for sustainability and motivation to sustain webMFT will be assessed during the semistructured interviews with survivors, coaches, and organizational staff (Table 3).

### Data Analyses

#### Overview

We will examine RE-AIM dimensions (Tables 1-3) to guide us in assessing the potential for larger-scale implementation of webMFT. We will conduct descriptive analyses of reach, adoption, and implementation outcomes including acceptability and feasibility of webMFT (eg, the number and mean duration of calls, barriers, and perceived feasibility of intervention delivery as judged by coaches). According to the current recommendations [46], we will consider webMFT feasible if >75% of the scheduled calls are delivered and survivor and coach and acceptability or satisfaction exceed 80% on rating scales (ratings of “moderately” to “very satisfied/acceptable”). Feedback from coaches and survivor debrief interviews will be used to revise and update webMFT for a larger-scale implementation trial.

We will assess potential between-group differences (webMFT vs MVPA tracking) in baseline variables (eg, age and medical history) using graphical methods, nonparametric and parametric tests as appropriate (eg, Wilcoxon rank-sum test for skewed data, 2-tailed *t* tests for normally distributed continuous data, and chi-squared tests for categorical data). Any variables not balanced by randomization will be controlled for as covariates in subsequent analyses, provided they are significantly associated with the outcome under consideration (at a modest *P*<.10 level).

We will assess effectiveness by examining the effects of webMFT versus MVPA tracking on the change in survivors’ MVPA in minutes per week (accelerometer data) at 12 weeks (3 months) using a mixed effects regression model, in which we will regress MVPA at 12 weeks on baseline MVPA, randomized group, wear time, and any variables not balanced by randomization. Models will include random intercepts to adjust for repeated measures within the participant over time. We will use a similar approach to estimate the effects of group on mood, QOL, physical function, and fatigue at 12 weeks. The interest is in estimating effect sizes and CIs. Analyses will be conducted on the intent-to-treat sample with all randomized participants included. Mixed effects models use a likelihood-based approach to estimation and do not require any direct imputation of missing outcomes.

#### Analyses of Qualitative Data

The audio-recorded interviews will be transcribed, and each transcript will be coded using a preliminary coding schedule. The coding scheme will be refined through an iterative process but will begin with the codes developed as a function of a priori research questions based on RE-AIM dimensions and PRISM domains. We will use NVivo software (Lumivero) to facilitate data coding and analyses. After data coding, we will identify themes across interviews [47] to develop a guide for future partnerships with organizations.

We will compare the relative cost-effectiveness of each arm in increasing survivors’ weekly MVPA minutes. Data collection following best practices for pragmatic measures will include tracking of the time, cost, and resources required to (1) recruit, train, and manage coaches; (2) recruit and enroll survivors; and (3) deliver the intervention. We will track Fitbit cost and delivery and Mentor1to1 technology–related costs [48]. Research-related activities and developmental costs will be excluded. Administrative time in (1)-(3) will be tracked automatically on Mentor1to1, reducing tracking time and administrative burden. We will assess costs to survivors, coaches, and organizations at the end of the study and conduct 3 separate analyses from the perspective of survivors, coaches, and organizations, respectively, to help prepare for implementation.

## Results

As of September 2023, phase 1 of the study was completed, and 61 survivors were enrolled in phase 2. We expect that using newer technologies for enhanced intervention delivery, program management, and automated data collection may facilitate effective implementation by organizations with limited resources. Adapting evidence-based MFT to a customized web platform and collecting data from coaches, survivors, and organizations along with costs will provide the basis for an implementation trial to increase MVPA and its benefits among many more survivors of breast cancer.

## Discussion

### Principal Findings

The 2 phases of this work address the adaptation of a web platform for an effective MVPA intervention (webMFT) followed by an RCT to test the effectiveness of webMFT as well as the RE-AIM outcomes of Reach, Adoption, Implementation and Maintenance that will inform future approaches to scale up this intervention. By adapting an existing peer mentoring web-based platform, Mentor1to1 for MFT, an intervention that was proven effective when delivered by phone, we expect to be able to scale up our intervention with fidelity and reduce the costs of intervention delivery. The benefits of this web-based platform include an efficient infrastructure to support coaches to deliver all aspects of the webMFT intervention, including key process steps to accept coaching requests, initiate secure phone calls, send text messages, and view survivor data. Administrative staff will use the web platform to manage the entire program (eg, make coach-survivor matches, review call recordings, and track Fitbit data). By using webMFT, we expect that coaches can reach many more survivors to become physically active, with potential health and psychosocial benefits [7,49,50].

This work is innovative because peer mentoring is relatively new in the PA literature, and to the best of our knowledge, our work in training cancer peer mentors (coaches) to support MVPA adoption in cancer survivors is unique. We plan to use user-centered iterative design for the adaptation and creation of webMFT, while retaining the same established principles and theories used in our previous work with additional technology-based enhancements. The updated web platform will streamline survivor and coach enrollment and will allow staff members to use real-time, multivariate matching between coaches and survivors. The current matching approach is manual, which is time-consuming and expensive. Our web platform–based intervention allows for the delivery of a multichannel (phone, SMS text messaging, and smartphone) peer mentoring approach in what we expect will be scalable, flexible, and low cost. All interactions will be recorded and tracked automatically through the platform, providing full oversight, detailed reports, and tracking of each coach’s time and effort. Finally, we expect that the web platform will integrate with a Fitbit activity tracker to provide automated MVPA data for the coach to review with the survivor during weekly calls. We expect that these features will reduce the need for investment in personnel to coordinate and collect data as well as facilitate intervention delivery.

Adapting our intervention to a web platform will require changes in the training program that we have provided the coaches. Our prior training required 4 in-person or videoconferencing group training sessions, each lasting about 2.5 hours. We will revise our training program extensively to include recorded training modules, a live internet-based group training focusing on training coaches to deliver the counseling content, and an individual training session to familiarize the coach with navigating the web platform while delivering the PA counseling. During the study implementation, we will also track any additional training and time spent by the research team to provide guidance and solve issues related to using the web platform. Careful attention to the changes required and adaptations in our training and supervision will allow the evidence-based intervention to be reliably implemented by cancer care organizations that have peer mentoring programs.

There is a large body of evidence to support the promotion of PA to facilitate the recovery and functioning of survivors of cancers, and numerous PA interventions (onsite, offsite, and hybrid) have been developed. Yet, these interventions have had limited applicability outside of research efforts. Much of the focus in implementing national PA guidelines for survivors of cancers [51-53] has been in health care settings. While there is considerable variation in the needs and challenges faced by survivors of cancers in adopting PA, there is a huge scope for community-based programs to meet the needs of those survivors who can safely become active outside of specialty care (eg, physical therapy and occupational therapy). There are numerous, well-established cancer support organizations in the United States focused on meeting the needs of their communities, especially patients or survivors, family members, and caregivers. Many of these organizations offer peer support programs. Our focus is to leverage the existing resources in these organizations, especially those which recognize the importance of PA in cancer recovery and make MFT more accessible to survivors.

To translate our research and build the capacity to improve survivors’ recovery, we do need to understand multilevel stakeholders’ perspectives on this partnership. By applying user-centered design principles and dissemination and implementation frameworks, we believe that we can design webMFT with dissemination and sustainability in mind from the beginning, thus improving the potential for adoption and sustainment. Key constructs measured with guidance from our expanded RE-AIM framework will inform future implementation efforts and improve our understanding of contextual factors at the levels of survivors, coaches, and organizations, which may influence future the adoption and maintenance of webMFT. Overall, these results will provide rich preliminary data to inform a future, large-scale pragmatic trial to involve multiple cancer care organizations in the delivery of webMFT to survivors of breast cancer across the country.

### Limitations

We have restricted the survivor sample to those who have smartphones. The use of smartphones has increased over time. We are designing for the future when the prevalence of communication technology will be higher; therefore, we anticipate that access will improve.

### Timeline

See Table 4 for the timeline and associated tasks.

**Table 4:** Study timeline.

Months		Study activity
1-48		Conduct weekly meetings with consultants (as needed) and research staff to plan, implement, problem solve, and review progress of the study Conduct weekly meetings with research staff to track progress, discuss and resolve problems, and plan all study activities
**Phase 1**		
	1-3	Hire study staff Obtain IRB^a^ approval
	4-6	Recruit 6-8 coaches from prior studies
	7-12	InquistHealth will adapt the web platform Mentor1to1 for MFT^b^ InquistHealth staff will work with coaches and obtain their feedback on webMFT^c^ Data collected (webMFT platform notes) and any written documentation will be stored at InquistHealth offices and their secure servers InquistHealth will modify and make changes in webMFT in response to feedback from coaches in an iterative approach
**Phase 2**		
	13-16	Work with collaborating organizations to recruit new coaches and survivors for the RCT^d^ to test webMFT
	16-18	Train new coaches (n=10-12) via videoconferencing Conduct posttraining evaluation of coaches
	17-30	Survivor recruitment and baseline assessments
	20-33	Intervention delivery
	24-33	Postintervention assessments of survivors
	34-36	Interviews with stakeholders at the collaborating cancer care organizations End of study interviews with coaches
	37-40	Transcription, coding, and thematic analyses of interview data (survivors, coaches, and stakeholder interviews)
	41-44	Develop a guide for developing and sustaining partnerships with cancer care organizations that have peer mentoring programs
	16-36	On-going data entry
	36-40	Quantitative analyses of data collected from coaches and survivors
	45-48	Manuscript preparation and begin plans for large-scale, pragmatic implementation trial

^a^IRB: Institutional Review Board.

^b^MFT: Moving Forward Together.

^c^webMFT: Mentor1to1 web platform adapted for Moving Forward Together.

^d^RCT: randomized controlled trial.

### Dissemination Plan

The study goals are to gather data to prepare for a larger-scale implementation trial of webMFT in several cancer care organizations that have peer mentoring programs. On a local and regional level, the work will be disseminated at the University of South Carolina–sponsored conferences, journal clubs, and community-based cancer support groups and local hospitals that have oncology programs. In addition, the results will be disseminated via presentations at regional, national, and international scientific meetings. A summary of the study results will be shared with the stakeholders at the organizations that have collaborated on the trial so that they can use the study results to inform their own staff, volunteers, cancer survivors, and potential funders.

### Conclusions

Survivors’ MVPA data from the RCT along with quantitative and qualitative data from coaches, survivors, and cancer organizations will provide the foundation for a large-scale implementation trial of the MFT program.

## Abbreviations

ACS: American Cancer Society
FRAME: Framework for Reporting Adaptations and Modifications-Enhanced
HIPAA: Health Insurance Portability and Accountability Act
MFT: Moving Forward Together
MVPA: moderate to vigorous physical activity
PA: physical activity
PRISM: Practical, Robust Implementation and Sustainability Model
QOL: quality of life
RCT: randomized controlled trial
RE-AIM: Reach, Effectiveness, Adoption, Implementation, Maintenance
REDCap: Research Electronic Data Capture
SCT: Social Cognitive Theory
TTM: Transtheoretical Model
webMFT: Mentor1to1 web platform adapted for Moving Forward Together

## Data Availability

Data sharing is not applicable to this article as no data sets were generated or analyzed during this study.
